# Changes of antibiotic prescribing pattern and its resistance to *E. Coli* in South Korea: a 12-year retrospective observational study

**DOI:** 10.1038/s41598-021-84450-z

**Published:** 2021-03-11

**Authors:** Geun Woo Lee, Sukhyun Ryu, Juhee Park, Eun Jee Lee, Kwang Jun Lee, Jungyeon Tae, Youngsik Hwang, Dong-Sook Kim

**Affiliations:** 1 Pharmaceutical and Medical Technology Research Team, Department of Research, Health Insurance Review and Assessment Service, Wonju, South Korea; 2 Department of Preventive Medicine, Konyang University College of Medicine, Daejeon, South Korea; 3 National Institute of Health, Korean Centers for Disease Control and Prevention, Osong, South Korea

**Keywords:** Health care, Medical research

## Abstract

In the present study, we investigated the pattern of changes in antibiotic prescription and antimicrobial resistance (AMR) in *Escherichia coli* in South Korea between 2007 and 2018. We collected data related to antibiotic prescription and AMR in *E. coli* from the national surveillance system. We used the Mann–Kendall test and Spearman’s correlation to identify the trends of antibiotic prescription and AMR in *E. coli* and to examine the relationship between them, respectively. Although we noted a significant decreasing trend of ampicillin and gentamicin prescriptions in all medical institutions, we identified a higher level of AMR in long-term care facilities than in other medical institutions. We did not identify a significant positive correlation between ampicillin and gentamicin prescriptions and their resistance in *E. coli*. However, we found a significant positive correlation between cefotaxime prescription and its resistance in *E. coli* in hospitals, long-term care facilities, and clinics. Our results strongly suggest that long-term care facilities in South Korea have the potential to sustain AMR epidemics and that more efforts are needed to curb AMR in *E. coli*. Further epidemiological studies using enhanced AMR surveillance are warranted.

## Introduction

The development of antimicrobial resistance (AMR) is a normal evolutionary process in microorganisms. It is often accelerated by selective pressure exerted by the overuse or misuse of antimicrobial agents^1^. The increasing use of antibiotics has led to the emergence of AMR, which limits the treatment of common bacterial infections. Gram-negative bacteria, such as *Escherichia coli*, which are resistant to third-generation cephalosporins, are particularly of great concern. This resistance is conferred by the production of extended-spectrum beta-lactamases (ESBLs) and is transferred to other gram-negative bacteria; therefore, the surveillance of AMR in *E. coli* is often used as a proxy for the surveillance of ESBLs in humans, animals, and the environment^2^.

South Korea is an East Asian country with a population of 51.4 million. In South Korea, broad-spectrum antibiotics, including quinolone, penicillin, and cephalosporin are frequently prescribed^3,4^. In the twenty-first century, extensive efforts have been made in South Korea to reduce AMR. These include the Korean National Antimicrobial Resistance Safety Control Program led by the Korean Ministry of Food and Drug Safety from 2003 to 2013^5^ and the Korean national action plan on AMR led by the Korean Ministry of Health and Welfare from 2016 to 2020^6^. To identify the level of antibiotic consumption and AMR within a country, population-based studies have mainly been conducted in countries in the UK and USA^7,8^. Different healthcare settings and public health policies in each country would affect the changes in antibiotic prescription because these factors are very likely to affect the selection pressures exerted on microorganisms in the population. Therefore, surveillance and research on changes in antibiotic consumption and AMR with changes in related health policies in different countries are important in order to develop evidence-based strategies to overcome AMR^6^. However, there exists a knowledge gap regarding the level of antibiotic prescription and AMR in Asia, which is highly vulnerable to AMR threats^9^.

To the best of our knowledge, no long-term observational study has been conducted in South Korea to assess the frequency of antibiotic prescription and AMR considering the changes in healthcare regulation. In the present study, we used the South Korean national surveillance data of 12 years to focus on broad-spectrum antimicrobial agents and their resistance in *E. coli*.

## Results

**Figure 1:**
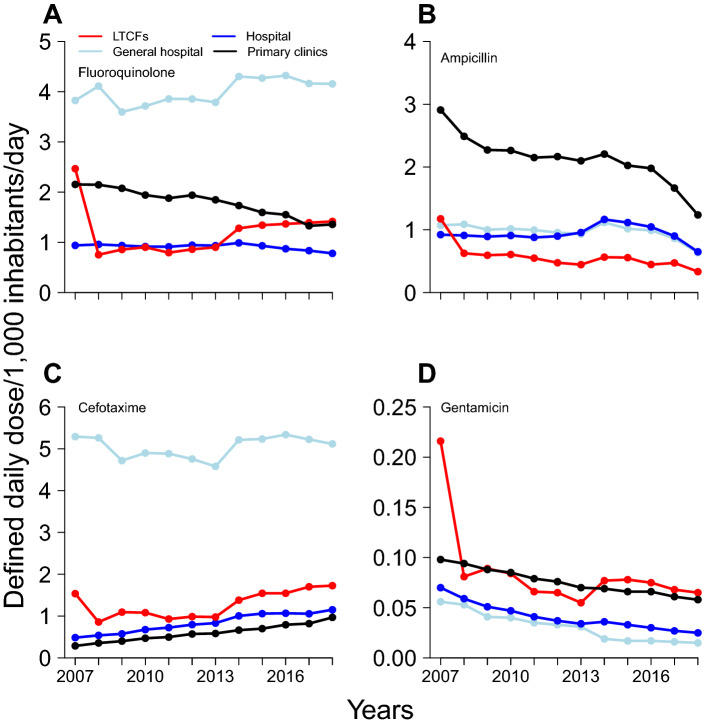
Prescription trend of antibiotics, including (**A**) fluoroquinolone, (**B**) ampicillin, (**C**) cefotaxime and (**D**) gentamicin, in South Korea between 2007 and 2018. The red line indicates long-term care facilities (LTCFs), and the blue line indicates hospitals. The white-blue line indicates general hospitals, and the black line indicates primary clinics.

The overall mean DID of fluoroquinolone and cefotaxime during the study period was 7.90 (range, 6.46–9.93) and 7.75 (6.21–9.19), respectively (Table 1). These antibiotics were prescribed more commonly in general hospitals than in other medical institutions. The overall mean DID of ampicillin and gentamicin was 4.60 (2.85–6.36) and 0.23 (0.15–0.44), respectively. Ampicillin was mostly prescribed in clinics (DID, 0.59; range, 1.24–2.91), while gentamicin was mostly prescribed in LTCFs (DID, 0.08; range, 0.06–0.22). We observed a decreasing trend of fluoroquinolone prescription in hospitals (τ =  − 0.52, *p* = 0.02) and clinics (τ =  − 0.94, *p* < 0.01) (Fig. 1, Supplementary Table 1). Furthermore, we observed a decreasing trend of ampicillin and gentamicin prescriptions in all medical institutions (Fig. 1, Supplementary Table 1). However, we noted an increasing trend of cefotaxime prescription in hospitals (τ = 0.94, *p* < 0.01), LTCFs (τ = 0.55, *p* = 0.02) and clinics (τ = 1.0, *p* < 0.01).

**Table 1:** Antibiotic prescription in South Korea between 2007 and 2018.

DID	General hospitals	Hospitals	LTCFs	Clinics	Overall
Fluoroquinolone	4.00 (3.60–4.32)	0.91 (0.78–0.99)	1.19 (0.75–2.47)	1.79 (1.33–2.15)	7.90 (6.46–9.93)
Ampicillin	0.97 (0.64–1.12)	0.94 (0.65–1.16)	0.57(0.33–1.18)	2.12 (1.24–2.91)	4.60 (2.85–6.36)
Cefotaxime	5.04 (5.34–4.58)	0.83 (0.49–1.15)	1.28 (0.86–1.73)	0.59 (0.29–0.97)	7.75 (6.21–9.19)
Gentamicin	0.03 (0.02–0.06)	0.04 (0.03–0.07)	0.08 (0.06–0.22)	0.08 (0.06–0.10)	0.23 (0.15–0.44)

Each value in the cell indicates the mean DID and the range (min–max).

*DID* defined daily dose/1000 inhabitants/day, *LTCFs* long-term care facilities.

**Figure 2:**
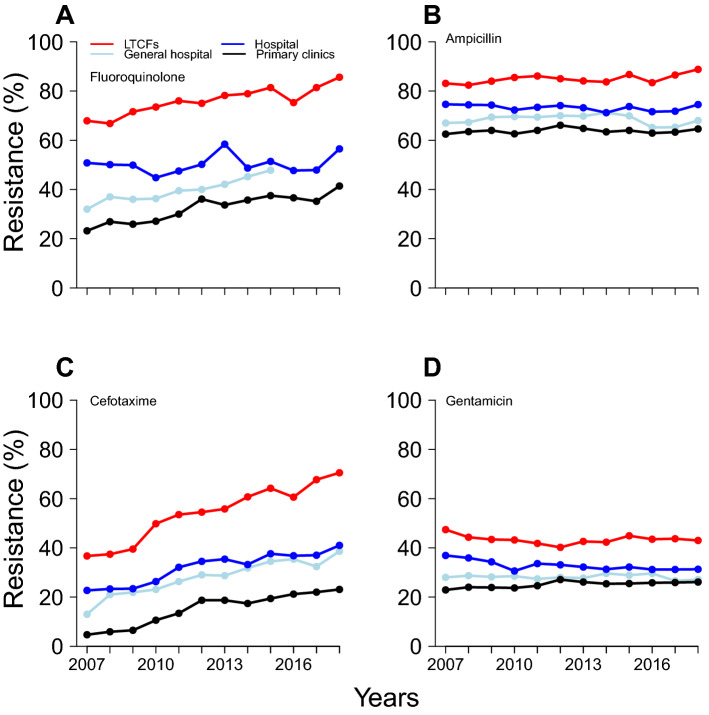
Resistance rate of *Escherichia coli* to antibiotics, including (**A**) fluoroquinolone, (**B**) ampicillin, (**C**) cefotaxime and (**D**) gentamicin, in South Korea between 2007 and 2018. The red line indicates long-term care facilities (LTCFs), and the blue line indicates hospitals. The white-blue line indicates general hospitals, and the black line indicates primary clinics.

The mean resistance rate of *E. coli* was the highest in LTCFs (fluoroquinolone 76%, ampicillin 85%, cefotaxime 54% and gentamicin 43%) (Table 2) and the lowest in clinics (fluoroquinolone 32%, ampicillin 64%, cefotaxime 15% and gentamicin 25%) (Table 2). We observed an increasing trend of fluoroquinolone resistance in *E. coli* in general hospitals (τ = 0.89, *p* < 0.01), LTCFs (τ = 0.81, *p* < 0.01) and clinics (τ = 0.76, *p* < 0.01). Moreover, we observed a significant increasing trend of cefotaxime resistance in *E. coli* in all medical institutions (Fig. 2, Supplementary Table 2). Furthermore, we identified increasing and decreasing trends of gentamicin resistance in *E. coli* in clinics (τ = 0.60, *p* < 0.01) and hospitals (τ =  − 0.64, *p* < 0.01), respectively.

**Table 2:** Antibiotic resistance rate of *Escherichia coli* in South Korea between 2007 and 2018.

Antibiotic to which *E. coli* was resistant	General hospitals	Hospitals	LTCFs	Clinics
Fluoroquinolone	39.54 (32.00–47.80)	50.33 (44.80–58.40)	75.97 (66.80–85.60)	32.44 (23.20–41.40)
Ampicillin	68.51 (65.20–71.20)	73.26 (71.20–74.60)	84.94 (82.40–88.80)	63.81 (62.50–66.10)
Cefotaxime	27.98 (13.00–38.60)	31.94 (22.70–41.00)	54.24 (36.770–70.50)	15.13 (4.70–23.10)
Gentamicin	28.19 (26.60–29.60)	32.82 (30.60–36.90)	43.36 (40.20–47.40)	25.08 (22.90–27.10)

Each value in the cell indicates the mean antimicrobial resistance rate and the range (min–max).

*LTCFs* long-term care facilities.

We observed a negative correlation between fluoroquinolone and gentamicin prescriptions and their resistance in *E. coli* in clinics (fluoroquinolone − 0.82, gentamicin − 0.73); however, we noted a positive correlation between gentamicin prescription and its resistance in *E. coli* in hospitals (τ = 0.69, *p* = 0.01) and LTCFs (τ = 0.65, *p* = 0.02) (Table 3). Moreover, we identified a positive correlation between cefotaxime prescription and its resistance in *E. coli* in hospitals (τ = 0.96, *p* < 0.01), LTCFs (τ = 0.66, *p* = 0.02) and clinics (τ = 0.98, *p* < 0.01).

**Table 3:** Correlation analysis of antibiotic prescription and resistance in *Escherichia coli* by the institutions between 2007 and 2018.

Antibiotic to which *E. coli* was resistant	General hospitals	Hospitals	LTCFs	Clinics
Fluoroquinolone	0.67 (0.06)	0.17 (0.60)	0.4 (0.15)	− 0.82 (< 0.01)
Ampicillin	0.24 (0.46)	− 0.46 (0.13)	− 0.53 (0.08)	− 0.31 (0.32)
Cefotaxime	0.13 (0.68)	0.96 (< 0.01)	0.66 (0.02)	0.98 (< 0.01)
Gentamicin	0.16 (0.61)	0.69 (0.01)	0.65 (0.02)	− 0.73 (< 0.01)

Each value in the cell indicates the coefficient and *p*-value.

*LTCFs* long-term care facilities.

## Discussion

The increasing use of antibiotics is a key factor contributing to the increased risk of AMR^10^. In the present study, we identified an increasing trend of cefotaxime prescription and of fluoroquinolone and cefotaxime resistance in *E. coli.* Furthermore, we identified a decreasing trend of fluoroquinolone prescriptions in hospitals and clinics and a decreasing trend of ampicillin and gentamicin prescriptions in all medical institutions.

A higher level of fluoroquinolone and cefotaxime was prescribed in general hospitals than in hospitals, which may be due to the severity of diseases in patients admitted there. Although fluoroquinolone and cefotaxime were more frequently prescribed in general hospitals, their resistance in *E. coli* was higher in LTCFs. This is likely because elderly patients usually visit general hospitals or hospitals for receiving medication rather than visiting LTCFs. Furthermore, AMR epidemics in LTCFs possibly affected the study results^11^.

The National Antimicrobial Resistance Safety Control Program implemented from 2003 to 2013 mainly included basic microbial research, AMR surveillance, and infection control (96% of the overall investment)^5^. However, this program could not extensively reduce AMR in *E. coli* during our study period.

The 5-year Korean action plan on AMR implemented in 2016 included the quality assessment of antibiotics used in medical institutions and improvement of the surveillance system of AMR^6^. The HIRA implemented a programme to assess the benefits of antibiotics prescribed for common cold during the study period and reported a 14% decrease in the number of antibiotics prescribed for common cold in clinics (from 54% in 2006 to 40% in 2017). This programme may largely reduce the prescription of beta-lactam antibiotics, which are the most commonly prescribed antibiotics in primary clinics in South Korea^4^. Furthermore, the large-scale public awareness campaign for the appropriate consumption of antibiotics may reduce the prescription of beta-lactam antibiotics in clinics^12^.

We identified a positive correlation between the prescription of fluoroquinolone and gentamicin and their resistance in *E.coli* in hospitals and LTCFs which is consistent with findings from other studies^13–15^. However, we identified a negative correlation in clinics. This result may be due to the decreasing trend of fluoroquinolone and gentamicin prescription in the clinic which was likely to be affected by the quality assessment program for antibiotic prescription in clinics from HIRA.

We also identified a negative correlation between ampicillin prescription and its resistance in *E. coli.* This result may be affected by the transmission of ESBLs, which confer resistance to third-generation cephalosporins, from *E. coli* and the resultant resistance to other beta-lactam antibiotics, including ampicillin^2,10^.

We found the highest AMR in LTCFs during our study period. Although antibiotic prescription is not very high in LTCFs in South Korea, most prescribed antibiotics are broad-spectrum, similar to the findings of a previous study^16^. Furthermore, AMR is higher in LTCFs than in the general community. This is in accordance with previous findings of higher AMR in elderly residents in LTCFs than in the general population^17,18^. This is likely due to the fact that most residents in LTCFs are elderly people with multiple chronic diseases and medications^19^ and with an increased risk of acquiring infections and developing AMR^20–22^. Very few studies have examined antibiotic prescription and resistance in LTCFs because of the characteristics of the resident population and the difficulty in forming a control group in the general population. Therefore, a study focusing on enhanced surveillance in LTCFs is needed to identify the risk factors for AMR and to measure the burden of AMR and the impact of AMR epidemic measures^11^.

There are several limitations of the present study. First, we did not include detailed data on demographic factors and comorbidities; therefore, we could not identify the risk factors for antibiotic prescription and AMR. Second, we did not include the appropriateness of antibiotic prescription, which would indicate the overuse of antibiotics. Third, we used DID as the indicator of antibiotic prescription rather than days of therapy or beds; however, this indicator has been widely used to monitor the trend of antibiotic prescription at the population level.

Fourth, we collected data regarding resistance in *E. coli* from the pathogen-based surveillance, which may limit the interpretation of AMR^23^. However, the present study provides insights into antibiotic prescription in South Korea, which may help in developing national antimicrobial stewardship. Enhanced surveillance of AMR, e.g. case-based surveillance, may overcome these limitations and help reveal the impact of AMR-related public health policies^23^.

In the present study, we identified a decreasing trend of ampicillin prescription but an increasing trend of cefotaxime prescription and cefotaxime resistance in *E. coli* between 2007 and 2018. Further research based on enhanced surveillance of AMR to measure the impact of AMR-related policies in South Korea is warranted.

## Methods

In this retrospective study, anonymised data regarding antibiotic prescription and AMR were extracted from the publicly available national database and laboratory-based surveillance system, respectively^24,25^. The study was approved and informed consent was waived by the Institutional Review Board of the Korean Health Insurance Review and Assessment Service (HIRA) (IRB No. 2017004-002). All methods were carried out in accordance with relevant guidelines from HIRA.

### Antibiotic prescription

We collected the data of antibiotic prescription from the nation-wide population-based database. The Korean National Health Insurance provides near-complete coverage of all antibiotic prescriptions in South Korea (approximately 98% of the total Korean population)^4^. All Korean medical institutions including general hospitals, hospitals, LTCFs, and clinics submit claims to the HIRA. The submitted medical claims include the name of the diagnosis, drug code, drug brand name, active ingredient, route of administration, amount in a single dose, daily dose, total number of days of administration or number of doses administered and prescription date. The administrative medical claims data collected from the HIRA were used for analysing the antibiotic prescriptions. The therapeutic drug class was determined using the World Health Organization’s Anatomical Therapeutic Chemical (ATC) classification system.

The antibiotics were classified into ATC code J01 (antibacterials for systemic use). Of the drugs registered between 2007 and 2018, a total of 3445 antibacterial agents were listed under ATC code J01. Analysis by class was performed for ATC levels 3 and 4, where level 3 corresponds to pharmacological and therapeutic properties and level 4 corresponds to chemical structures.

To measure the standardised level of antibiotic prescription across various antibiotic products, annual antibiotic prescriptions were calculated as the defined daily dose (DDD)/1000 inhabitants/day. This indicator was widely used to examine the relation between antibiotic prescription and AMR on population-level^26,27^. The annual population between 2007 and 2018 was acquired from Statistics Korea^28^.

$$ {\text{DDD}}/1000\;{\text{inhabitants}}/{\text{day}} = \frac{{\sum Xij \left( {{\text{mg}}} \right) \times 1000\;{\text{persons}}}}{{{\text{DDDi }}\left( {{\text{mg}}} \right) \times 365 \times {\text{Pi}}}} $$

X: drug use, P: number of people, i: population category (gender, age and facilities), j: ATC.

### Antimicrobial resistance

We collected the resistance rate from the Korean Antimicrobial Resistance Monitoring System (KARMS) operated by the Korea Centers for Disease Control and Prevention (KCDC)^29^. This national AMR monitoring system is laboratory-based sentinel surveillance which collected laboratory data from the KCDC designated hospitals and laboratories where performed KCDC approved antimicrobial susceptibility tests^30^. In South Korea, the physicians in the primary clinics and LTCFs usually examined the clinical samples of their patients at the KCDC designated laboratories. The sentinel institutions including hospitals and laboratories report to KARMS the AMR rate which was calculated using the arithmetic mean values of the clinical sample obtained from hospitals, LTCFs, and clinics. This sentinel surveillance of AMR has been widely used for tracking the proportions of resistant isolates in the country level^31^. From 2016, the KCDC-designated laboratory institutions examined the specimens collected from the sentinel institutions.

### Definitions

General hospitals and hospitals were defined as medical institutions with more than 100 beds and 30–100 beds, respectively. LTCFs were defined as institutions that mainly provides rehabilitative care for the elderly. Clinics were defined as institutions with less than 30 beds that mainly provided outpatient care^25^.

### Statistical analysis

We used the Mann–Kendall test to identify the trends of antibiotic prescription and AMR in *E. coli* and used Spearman’s correlation to examine the relationship between them. In the present study, we excluded the prescription data of 2007 from long-term care facilities (LTCFs), as some institutions had outlier data. Moreover, fluoroquinolone resistance rates of *E. coli* were not measured in general hospitals from 2016 to 2018. Analyses were performed using R version 3.6.1 (R Foundation for Statistical Computing, Vienna, Austria).

### Ethics approval

The present study was approved by the Institutional Review Board of the Korean Health Insurance Review and Assessment Service (HIRA) (IRB No. 2017004-002). The informed consent was waived by the Institutional Review Board of HIRA.

## Supplementary Information

Supplementary Information 1.

## Data Availability

All data used within the study are available from the corresponding author on request.
