# Supplementary material for: Changes of antibiotic prescribing pattern and its resistance to E. Coli in South Korea: a 12-year retrospective observational study
Source: Sci Rep. 2021 Mar 11;11:5658. doi: 10.1038/s41598-021-84450-z (PMC7970963; doi:10.1038/s41598-021-84450-z)
Supplement: Supplementary file 1 — Supplementary Information 1. [file 41598_2021_84450_MOESM1_ESM.docx]

**Supplementary data file**

Changes of antibiotic prescribing pattern and its resistance to *E. Coli* in South Korea: A 12-year retrospective observational study

Geun Woo Lee^1*^, Sukhyun Ryu^2*^, Juhee Park^1^, Eun Jee Lee^1^, Kwang Jun Lee^3^, Jungyeon Tae^2^, Youngsik Hwang^2^, Dong-Sook Kim^1^

^1^Pharmaceutical & Medical Technology Research Team, Department of Research, Health Insurance Review & Assessment Service, Wonju, South Korea

^2^Department of Preventive Medicine, Konyang University College of Medicine, Daejeon, South Korea

^3^National Institute of Health, Korean Centers for Disease Control and Prevention, Osong, South Korea

*Co-first authors

Corresponding author: sttone@hira.or.kr

Supplementary Tables.

Supplementary Table 1. Trend analysis of antibiotic consumption by the institutions between 2007 and 2018. Each value in the cell indicates the coefficient and p-value.

| DID | General hospitals | Hospitals | LTCFs | Clinics |
| --- | --- | --- | --- | --- |
| Fluoroquinolone | 0.42 (0.06) | −0.52 (0.02) | 0.55 (0.16) | −0.94 (<0.01) |
| Ampicillin | −0.53 (0.02) | 0 (1) | −0.70 (<0.01) | −0.88 (<0.01) |
| Cefotaxime | 0.03 (0.95) | 0.94 (<0.01) | 0.55 (0.02) | 1 (<0.01) |
| Gentamicin | −0.99 (<0.01) | −0.97 (<0.01) | −0.50 (0.03) | −0.99 (<0.01) |

DID: defined daily dose/1,000 inhabitants/day; LTCFs: long-term care facilities

Supplementary Table 2. Trend analysis of antibiotic resistance in *Escherichia coli* by the institutions between 2007 and 2018. Each value in the cell indicates the coefficient and p-value.

| Antibiotic to which *E. coli* was resistant | General hospitals | Hospitals | LTCFs | Clinics |
| --- | --- | --- | --- | --- |
| Fluoroquinolone | 0.89 (<0.01) | 0.06 (0.84) | 0.81 (<0.01) | 0.76 (<0.01) |
| Ampicillin | 0.11 (0.68) | −0.36 (0.11) | 0.42 (0.06) | 0.17 (0.49) |
| Cefotaxime | 0.91 (<0.01) | 0.88 (<0.01) | 0.94 (<0.01) | 0.93 (<0.01) |
| Gentamicin | −0.09 (0.73) | −0.64 (<0.01) | −0.15 (0.54) | 0.60 (<0.01) |

LTCFs: long-term care facilities
